# Network meta-analysis comparing neoadjuvant chemoradiation, neoadjuvant chemotherapy and upfront surgery in patients with resectable, borderline resectable, and locally advanced pancreatic ductal adenocarcinoma

**DOI:** 10.1186/s13014-019-1330-0

**Published:** 2019-07-10

**Authors:** Qiancheng Hu, Dan Wang, Ye Chen, Xiaofen Li, Peng Cao, Dan Cao

**Affiliations:** 1 Department of Abdominal Oncology, Cancer Center, West China Hospital, Sichuan University, No. 37 Guo Xue Xiang, Chengdu, 610041 China; 2 Department of Medical Oncology, Cancer Center, West China Hospital, Sichuan University, Chengdu, China

**Keywords:** Pancreatic ductal adenocarcinoma, Neoadjuvant therapy, Bayesian analysis, Network meta-analysis

## Abstract

**Purpose:**

Neoadjuvant chemoradiation or chemotherapy has improved the treatment efficacy of patients with resectable, borderline resectable, and locally advanced pancreatic ductal adenocarcinoma (PDAC). Due to the optimal regimen remains inconclusive, we aimed to compare these treatments in terms of margin negative (R0) resection rate and overall survival (OS) with Bayesian analysis.

**Patients and methods:**

We reviewed literature titles and abstracts comparing three treatment strategies (neoadjuvant chemoradiation, neoadjuvant chemotherapy, and upfront surgery) in PubMed, Embase, Cochrane Library, the American Society of Clinical Oncology and ClinicalTrials.gov database from 2009 to 2018 to estimate relative odds ratios (ORs) for margin negative (R0) resection rate and hazard ratios (HRs) for overall survival (OS) in all include trials.

**Results:**

A total of 14 literatures with 1056 patients were enrolled in this Bayesian analysis. In the pairwise meta-analysis from limited head-to-head studies, compared with neoadjuvant chemotherapy, neoadjuvant chemoradiation showed superior OS significantly (HR 0.8, 95% CI 0.60–0.99, *p* < 0.001*)* and there was no significant difference in R0 resection rate (OR 1.02, 95%CI 0.45–2.33, *I*^2^ = 34.6%). However, in the network meta-analysis from all enrolled clinical trials, neoadjuvant chemoradiation showed significantly higher R0 resection rate over upfront surgery (HR 0.15, 95% CrI 0.02–0.56), whereas neoadjuvant chemotherapy did not provide better efficacy in R0 resection over upfront surgery (HR 0.42, 95% CrI 0.02–4.41). For R0 resection rate, neoadjuvant chemoradiation has the highest probability of ranking one compared with neoadjuvant chemotherapy or upfront surgery (79% vs 21% vs 0%). For OS, neoadjuvant chemotherapy has the highest probability of ranking one compared with neoadjuvant chemoradiation or upfront surgery (98% vs 0% vs 2%). Neoadjuvant chemotherapy was associated with higher rates of postoperative complications (rank worst: 84%), followed by neoadjuvant chemoradiotherapy (13%) and upfront surgery (3%).

**Conclusions:**

Different neoadjuvant treatment was selected based on various purposes, whether increasing R0 resection rate or not. Future clinical trials comparing neoadjuvant chemoradiation with neoadjuvant chemotherapy are warranted to confirm our results.

## Introduction

Pancreatic ductal adenocarcinoma (PDAC) is one of the most lethal cancers in the world [1]. Prognosis is dismal, and the 5-year survival rate is within 5% [2]. Radical resection with a negative margin, such as margin-negative (R0) resection, is the key point for long-term survival of this aggressive malignancy [3].

Since 2009, local pancreatic ductal adenocarcinoma has been broadly classified into three categories: resectable, borderline resectable and locally advanced disease based on vascular involvement assessed by preoperative imaging in the expert consensus [4, 5]. In general, approximately 10–20% of PDCA patients are present with surgically resectable disease [2], while 30–40% of patients are present with “borderline resectable pancreatic adenocarcinoma (BRPC)” or “locally advanced pancreatic adenocarcinoma (LAPC)”. These patients have a low R0 resection rate and high potential of R1, making them theoretically the ideal candidates for neoadjuvant therapy [6].

It is becoming more obvious that patients with BRPC, who are at a higher risk for R1 resection, are potentially in need of neoadjuvant therapy with the goal of improving overall survival (OS) [7, 8]. According to a systematic review and meta-analysis of 19 cohort studies, patients with unresectable disease (BRPC and LAPC) who had undergone neoadjuvant therapy had similar survival outcomes as the patients who were initially deemed resectable pancreatic adenocarcinoma (RPC) [9]. Dhir et al. evaluated the effects of neoadjuvant therapy including 5520 patients with local PDAC and reported that neoadjuvant therapy demonstrated OS benefit (HR 0.58,95%CI 0.46–0.70), without increasing grade ≥ 3 toxicities (OR 0.36, 95%CI 0.24–0.48) [10]. Neoadjuvant therapy was also applied in managing patients with RPC sometimes, especially for patients with high-risk features. The systematic review confirmed that neoadjuvant treatment appeared to improve the OS by intention to treat, although showing lower overall resection rates for RPC or BRPC [11].

The studies mentioned above addressed the effectiveness and safety of neoadjuvant therapies involving neoadjuvant chemotherapy, or chemoradiation compared with upfront surgery in patients with local PDAC. However, they did not focus on the advantage between neoadjuvant chemoradiation and neoadjuvant chemotherapy, and there were very limited data available for head to head research comparing the main neoadjuvant regimens. Furthermore, to the best of our knowledge, no literature has reported a comprehensive comparison of three methods (neoadjuvant chemoradiation vs neoadjuvant chemotherapy vs upfront surgery). This study compared these methods for the treatment of resectable, borderline resectable, and locally advanced PDAC comprehensively by applying a network meta-analysis, with an expectation to provide some reference for selecting appropriate treatment.

## Methods

### Search strategy and selection criteria

The Bayesian analysis was conducted and reported in accordance with the PRISMA (Preferred Reporting Items for Systematic Reviews and Meta-Analyses) guidelines. We searched PubMed, Embase (Ovid), Cochrane Library, the American Society of Clinical Oncology and ClinicalTrials.gov database using “neoadjuvant therapies of pancreatic ductal adenocarcinoma” as part of the titles and abstracts with English restrictions. A time-frame from January 1, 2009, which was the date of introducing local anatomic subcategories of pancreatic ductal adenocarcinoma, i.e., RPC, BRPC and LAPC [4, 5], to December 16, 2018, was selected for the database search. In addition, we manually searched related reviews and bibliographies of included trials for additional references.

References were included if: 1. local PDAC; 2. compare two of the three treatment strategies (neoadjuvant chemoradiation, neoadjuvant chemotherapy, upfront surgery) with each other; 3. report enough information to calculate hazard ratios (HRs); 4. unrestricted age, gender, performance status (PS), ethnicity and country.

The references were excluded according to the following criteria: 1. neoadjuvant targeted therapy; 2. neoadjuvant chemoradiation and chemotherapy mixed; 3. posters and abstracts; 4. single-arm studies.

The search strategy in strict accordance with Population Intervention Comparison Outcomes Study (PICOS) design framework included the following domains of Medical Subject Heading (MeSH) terms: ‘Pancreatic Neoplasms’ and ‘Neoadjuvant Therapy’; MeSH and Subheadings were combined with ‘AND’ or ‘OR’.

### Data extraction and assessment for bias risk

First, the titles and abstracts of articles were screened. Review articles, case series, case reports, guidelines and conference abstracts were excluded from our study; and then full-text articles that met the inclusion criteria were thoroughly reviewed. Two investigators (Qc H, D W) reviewed the full manuscripts independently and extracted information including patient characteristics, period and type of study, treatment protocols, the sample size and outcomes (median OS, hazard ratio, 95% confidence interval and R0 resection rate) into the electronic database. Disagreements in study and data selection among investigators were resolved by discussion and consensus. The quality and risk of bias of randomized controlled trials (RCTs) were assessed by using Cochrane Collaboration’s tool [12], and the other trials were assessed by Risk if Bias in Non-randomized Studies of Interventions (ROBINS-I) [13].

### Data synthesis and analysis

The outcomes we analysed were median OS and R0 resection rate. R0 was defined as margin negative if tumour cells were present > 1 mm from the any surface. R1 was defined as margin positive if tumour cells were present within 1 mm from the any surface [14, 15]. Results on OS in the Bayesian analysis were expressed as a hazard ratio (HR) with 95% confidence intervals (CI). *P* < 0.05 was considered as significant level. Heterogeneity was assessed with the *I*^2^ statistic. *I*^2^ values less than 25% and greater than 50% were regarded as indicating low and high heterogeneity, respectively [16]. When HRs were not reported, we made estimations from summary statistics with the method described by Tierney et al. in 2007 [17], and Kaplan-Meier curves were digitized using Getdata Graph Digitizer 2.26 (http://www.getdata-graph-digitizer.com). We applied the traditional pairwise meta-analysis between direct comparisons with Stata13 (StataCorp, College Station, TX, USA). The network meta-analysis was conducted with GeMTC version 0.14.3 (http://drugis.org/software/addis1/gemtc) and WinBUGS version1.4.3 (MRC Biostatistics Unit, Cambridge, UK) by fixed-effect models. For R0 resection rate, we used GeMTC for network meta-analysis. Parameters for the GeMTC software were selected as: number of chains, 4; tuning iterations, 20,000; simulation iterations, 50,000; thinning interval, 10; inference samples, 10,000; and variance scaling factor, 2.5. Besides, we chose 5000 burn-ins and a thinning interval of 1 for each chain for HRs. The consistency model would be used when there was no significant inconsistency; otherwise, the inconsistency model was applied. We assessed the convergence of the model using the potential scale reduction factor (PSRF) of the Brooks–Gelman–Rubin method [18]; PSRF closer to 1 indicated the better convergence.

## Results

### Eligible studies and characteristics

**Fig. 1:**
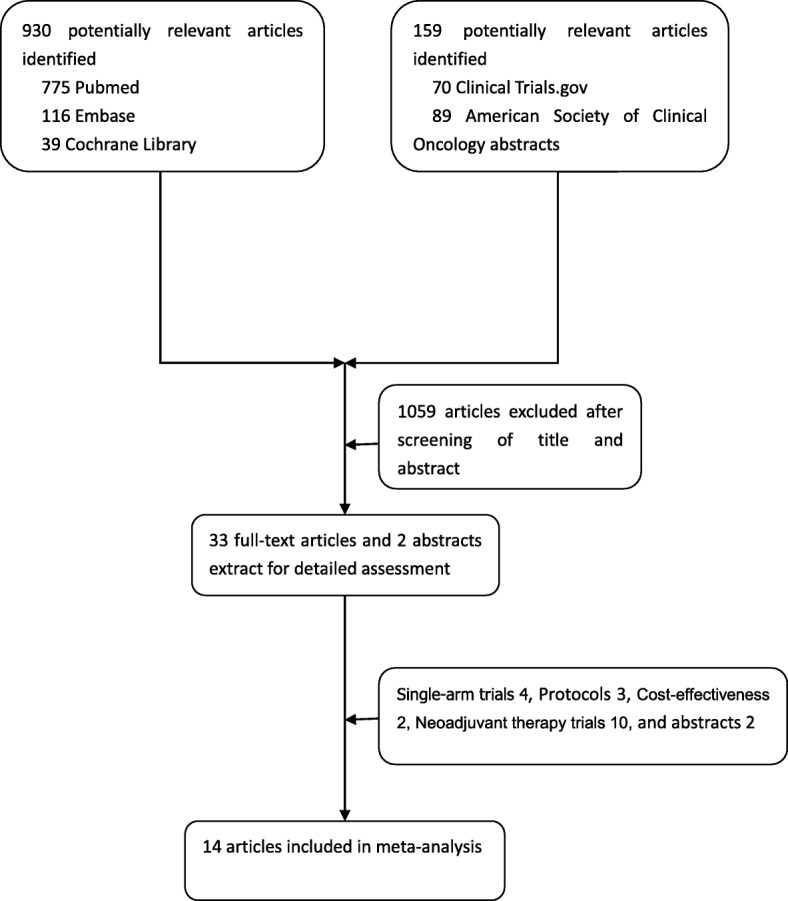
Literature search and selection

We identified 1089 studies from the title and abstract review to start with (Fig. 1). After initial screening, we retrieved the full text of potentially eligible articles for further detailed assessment. With the predeveloped search strategy, 14 eligible publications including three randomized controlled trials were included for meta-analysis, with a total of 1056 patients received at least one of the three treatment strategies (Table 1) [19–32]. The eligible studies were published during 2010 to 2018. All studies included in our Bayesian analysis have been published as full manuscripts.

**Table 1:** Main characteristics of the controlled trials included in the meta-analysis

References	Type of study	Disease	Period of study	Intervention regimen	Control regimen	Participants	Median OS	HR (95% CI)	R0 (%)
Hackert et al.	Retrospective	LAPC	2001–2015	Chemoradiotherapy	Chemotherapy	322 vs 125	16.5 vs 16	0.93(0.67–1.28)	31.3% vs 40.8%
Shrestha et al.	Retrospective	BRPC	2007–2012	Chemoradiotherapy	Chemotherapy	19 vs 14	16.4 vs 10.9	0.75(0.38–1.51)	NA
Kim et al.	Retrospective	BRPC	2007–2015	Chemoradiotherapy	Chemotherapy	25 vs 15	20.1 vs 16.1	0.67(0.26–1.72)	NA
Satoi et al.	Prospective	BRPC/LAPC	2008–2013	Chemoradiotherapy	Chemotherapy	35 vs 32	22 vs Not reached	0.53(0.27–1.02)	91% vs 81%
Lloyd et al.	Retrospective	BRPC/LAPC	2000–2013	Chemoradiotherapy	Chemotherapy	23 vs 65	12.5 vs 13.9	1.09(0.65–1.82)	9% vs 6%
Barbier et al.	Retrospective	LAPC	1997–2006	Chemoradiotherapy	Surgery-first	88 vs 85	21.5 vs 18	1.16(0.7–1.91)	92% vs 67%
Casadei et al.	RCT	RPC/BRPC/LAPC	2007–2014	Chemoradiotherapy	Surgery-first	18 vs 20	22.4 vs 19.5	NA	38.9% vs 25%
Casadei et al.	Observtional	BRPC	2000–2013	Chemoradiotherapy	Surgery-first	30 vs 28	NA	NA	93.3 vs 71.4%
Fujii et al.	Observtional	BRPC	2002–2014	Chemoradiotherapy	Surgery-first	21 vs 71	29.1 vs 13.1	0.28(0.10–0.75) ^a^	100% vs 40%
Fujii et al.	Prospective	RPC/BRPC	2001–2013	Chemoradiotherapy	Surgery-first	40 vs 233	28.6 vs 33.7	0.79(0.28–2.22)	86% vs 70%
Jang et al.	RCT	BRPC	2012–2014	Chemoradiotherapy	Surgery-first	27 vs 23	21 vs 12	0.53(0.29–0.98)	82.4% vs 33.3%
Tafima et al.	Retrospective	RPC	2006–2009	Chemotherapy	Surgery-first	13 vs 21	NA	1.18(0.43–3.24)	84.6% vs 85.7%
Golcher et al.	RCT	RPC	2003–2009	Chemoradiotherapy	Surgery-first	33 vs 33	17.4 vs 14.4	0.96(0.55–1.67)	89.5% vs 69.6%
Murakami et al.	Retrospective	BRPC	2002–2015	Chemotherapy	Surgery-first	52 vs 25	27.1 vs 11.6	0.42(0.25–0.72)	72.3% vs 17.4%

^a^ Data extraction from article; *RCT* Randomised Controlled Trial, *LAPC* Locally Advanced Pancreatic Cancer, *BRPC* Borderline Resectable Pancreatic Cancer, *RPC* Resectable Pancreatic Cancer, *NA* Not answer

### Pairwise meta-analysis for OS and R0 resection rate

**Fig. 2:**
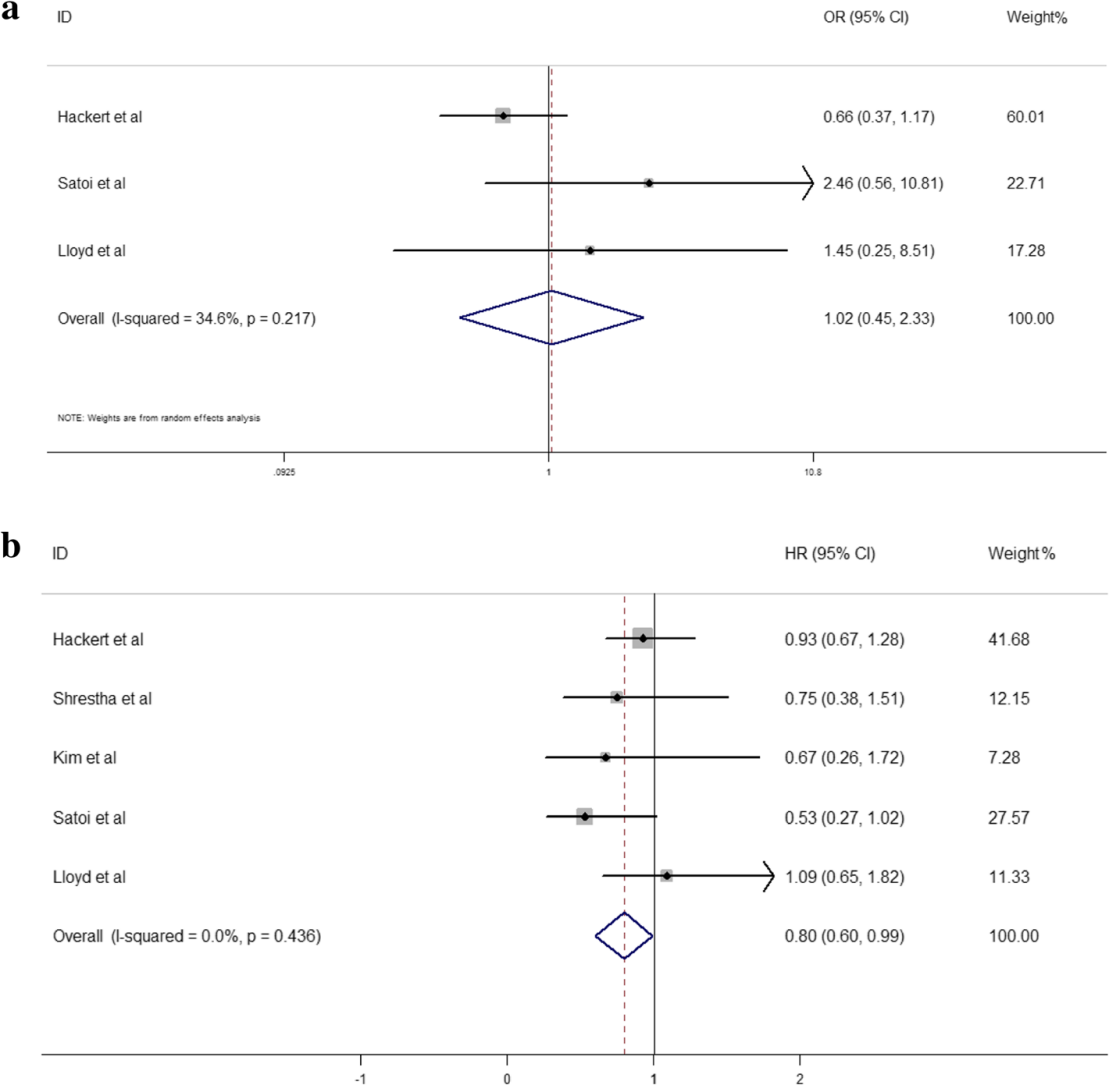
**a** Comparison of R0 resection rate according to pairwise meta-analysis. **b** Comparison of overall survival according to pairwise meta-analysis

There were five head-to-head studies which compared the OS between neoadjuvant chemoradiation and neoadjuvant chemotherapy. Within the five head-to-head studies, only three of them reported the R0 resection rate. In our pairwise meta-analysis, there was no significant difference between neoadjuvant chemoradiation and neoadjuvant chemotherapy in R0 resection rate (OR 1.02, 95%CI 0.45–2.33, *I*^2^ = 34.6%, Fig. 2a). Neoadjuvant chemoradiation showed superior OS significantly when compared with neoadjuvant chemotherapy (HR 0.8, 95% CI 0.60–0.99, *p* < 0.001, Fig. 2b).

### Networks for multiple treatment comparisons

**Fig. 3:**
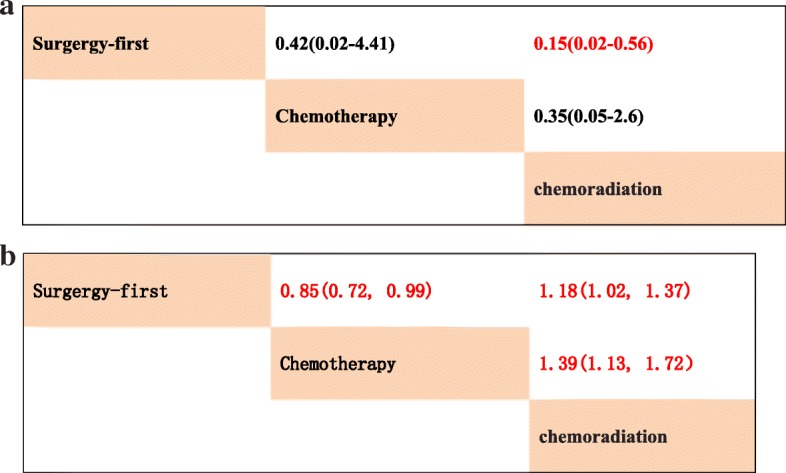
**a** Pooled odds ratios for R0 resection rate. (Red represents statistical significance). **b** Pooled hazard ratios for overall survival. (Red represents statistical significance)

The network was designed for three treatment comparisons of neoadjuvant chemoradiation, neoadjuvant chemotherapy and upfront surgery. All the potential scale reduction factors (PSRFs) were in the range of 1.00 to 1.01, so the model was proven convergent and stable; the consistency model was adopted. According to the established network, based on the consistency model, neoadjuvant chemoradiation showed significantly higher R0 resection rate over upfront surgery (HR 0.15, 95% CrI 0.02–0.56), whereas neoadjuvant chemotherapy did not provide better efficacy in R0 resection over upfront surgery (HR 0.42, 95% CrI 0.02–4.41) (Fig. 3a). Compared with results from the traditional pairwise meta-analysis, neoadjuvant chemotherapy showed a superior advantage for OS over neoadjuvant chemoradiation (HR 0.72, 95% CrI 0.58–0.88) or upfront surgery (HR 0.85, 95% CrI 0.72–0.99) in network meta-analysis (Fig. 3b).

**Fig. 4:**
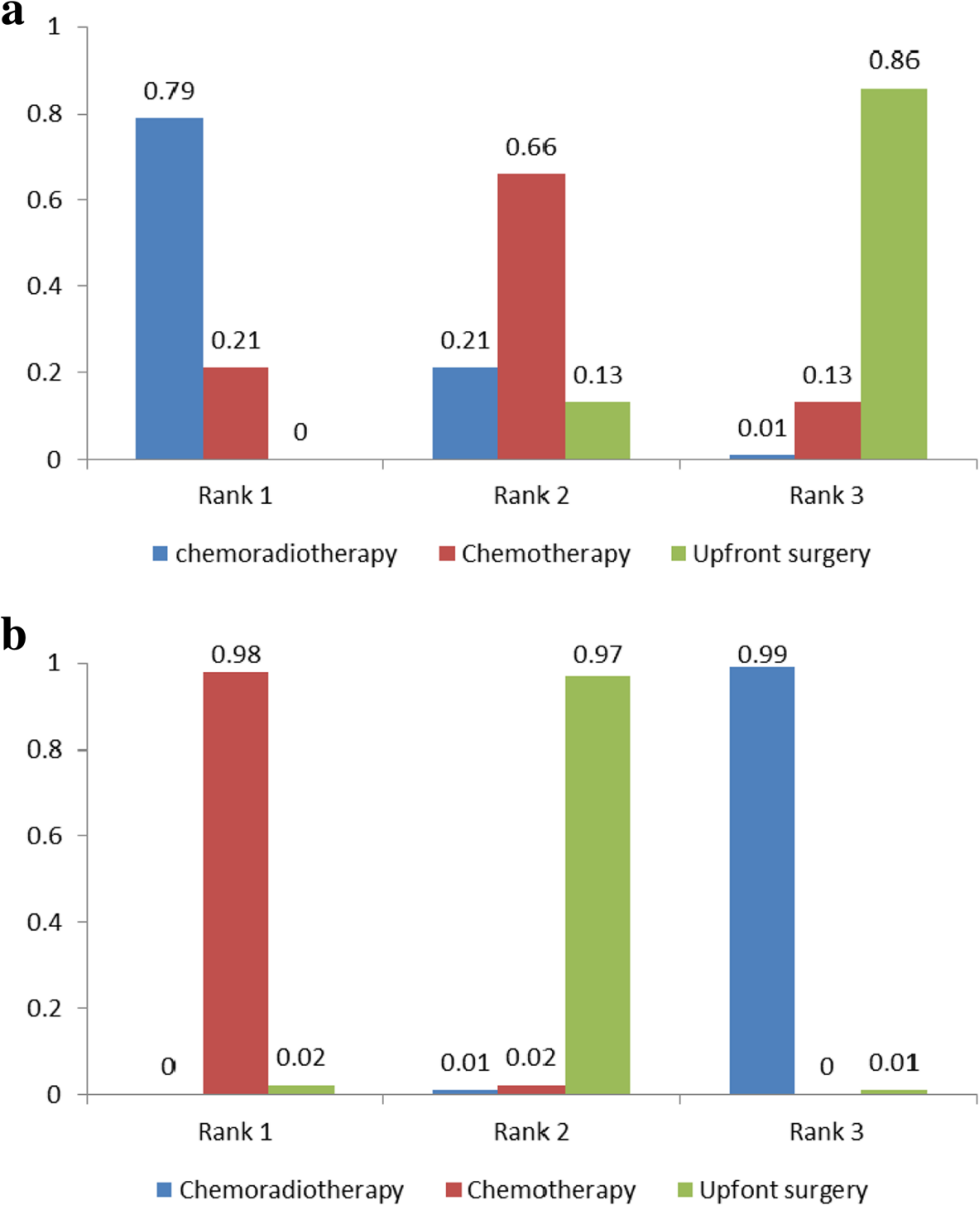
Ranking of treatments in terms of R0 resection and overall survival

The rank probabilities, calculated by the network consistency model, ranked the probabilities of rank order of treatment regimen overall outcomes evaluated (neoadjuvant chemoradiation, neoadjuvant chemotherapy and upfront surgery). Regimens with a higher value in the histogram were associated with higher probabilities for better treatment outcomes. The probability distribution of each regimen which was ranked at each of the possible positions was showed by the histogram (Fig. 4a-b). For the R0 resection rate, neoadjuvant chemoradiation had the highest probability of ranking one compared with neoadjuvant chemotherapy or upfront surgery (Fig. 4a, 79% vs 21% vs 0%). For OS, neoadjuvant chemotherapy had the highest probability of ranking one compared with neoadjuvant chemoradiation or upfront surgery (Fig. 4b). Neoadjuvant chemoradiation ranked as highest (98% vs 0% vs 2%) among all the regimens in all R0 resection rates measured without improving the likelihood of OS in local PDAC.

**Fig. 5:**
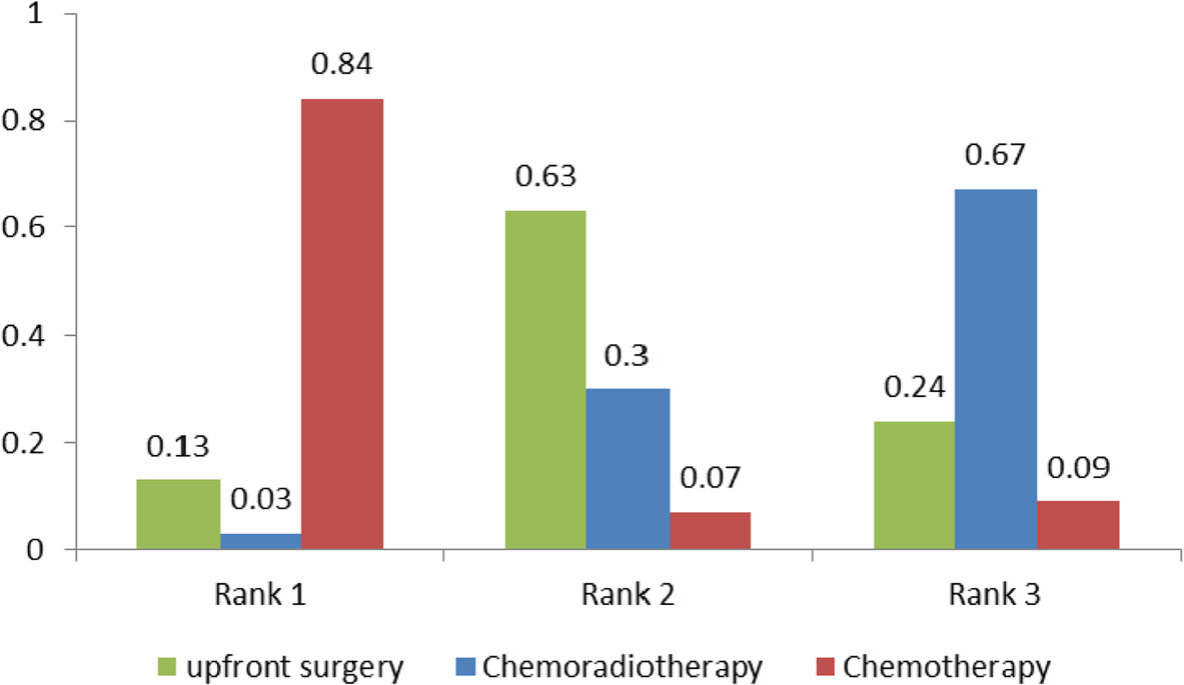
Ranking of treatments in terms of postoperative complications

The comparison of postoperative complications among different treatment strategies was carried out. None of the five studies provided data on postoperative complications. The rankings of the three competitive treatment strategies were summarised in terms of postoperative complications (Fig. 5). Regimens with a higher value in the histogram were associated with higher probabilities for worse treatment outcomes. Neoadjuvant chemotherapy was associated with higher rates of postoperative complications (rank worst: 84%), followed by neoadjuvant chemoradiotherapy (13%) and upfront surgery (3%).

## Discussion

Our study compared neoadjuvant chemoradiation, neoadjuvant chemotherapy and upfront surgery for local PDAC including both R0 resection rate and OS by using pairwise meta-analysis and network meta-analysis. In our pairwise meta-analysis, there was no significant difference between neoadjuvant chemoradiation and neoadjuvant chemotherapy in R0 resection rate, with only three studies reporting the data. However, in this network analysis, our results suggested that neoadjuvant chemoradiation provided R0 resection rate advantage over upfront surgery in indirect evidence and had the highest probability of ranking one compared with neoadjuvant chemotherapy or upfront surgery. In general, neoadjuvant chemoradiation might be the most popular choice of treatment for local PDAC, especially for BRPC and LAPC.

In our study, neoadjuvant chemoradiation provided an OS advantage over neoadjuvant chemotherapy suggested by the pairwise meta-analysis, but not that of network meta-analysis likely because the enrolled patients could not be subgrouped, such as RPC, BRPC and LAPC. Zhan et al. proved that neoadjuvant therapy would be beneficial for patients with BRPC and LAPC in the meta-analysis of prospective studies, but neoadjuvant chemoradiation did not achieve a better prognosis than neoadjuvant chemotherapy alone (median OS: 16.7 months vs 16.8 months) [33]. As more than 75% of patients received neoadjuvant radiotherapy in the meta-analysis [34], controversies still existed, and it was difficult to achieve consensus on applying neoadjuvant chemoradiation and chemotherapy in the management of local PDAC.

Interestingly, Schorn et al. addressed that neoadjuvant therapy reduced the risk of local recurrence (RR 0.42, 95%CI 0.32–0.55) but not the risk of distant metastasis (RR 1.02, 95%CI 0.91–1.14) in BRPC and LAPC in the meta-analysis of observational studies [34]. As the majority (91.7%) of studies included neoadjuvant chemoradiation, it perhaps, be an effect of neoadjuvant chemoradiation on low local recurrence rate for local PDAC. This result illustrated neoadjuvant chemoradiation might be the most popular choice of treatment for local PDAC indirectly, and it was in accordance with our study. Subsequent therapies are another questionable factors for different results of direct and indirect meta-analysis [35].

Our study also had several limitations. First, although there was no obvious inconsistency and severe risk of bias detected, our study only enrolled three RCTs with a relatively small number of participants that might weaken the evidence of our meta-analysis. Second, given the dissimilar disease of participants with RPC, BRPC and LAPC, further subgroup analysis was not conducted. Lastly, different (neo) adjuvant chemotherapy regimens might had an effect on the OS.

## Conclusions

In conclusion, according to the comprehensive evaluation suggested by Bayesian analysis, neoadjuvant chemoradiation provided better R0 resection rate advantage compared with neoadjuvant chemotherapy and upfront surgery, and neoadjuvant chemotherapy improved OS for local PDAC. Selection of different neoadjuvant therapy will reply on treatment purpose of whether need increase R0 resection rate or not. Additional head to head clinical data that further define the long-term efficacy of neoadjuvant chemoradiation and chemotherapy are needed to confirm our results.

## Data Availability

This Bayesian analysis used data from PubMed, Embase (Ovid), Cochrane Library, the American Society of Clinical Oncology and ClinicalTrials.gov database.

## Abbreviations

AEs: Adverse events
BRPC: Borderline resectable pancreatic adenocarcinoma
CI: Confidence intervals
HRs: Hazard ratios
LAPC: Locally advanced pancreatic adenocarcinoma
MeSH: Medical Subject Heading
ORs: Relative odds ratios
OS: Overall survival
PDAC: Pancreatic ductal adenocarcinoma
PICOS: Population Intervention Comparison Outcomes Study Design
PRISMA: Preferred Reporting Items for Systematic Reviews and Meta-Analyses
PSRFs: Potential scale reduction factors
ROBINS-I: Risk Of Bias In Non-randomised Studies of Interventions
RPC: Resectable pancreatic adenocarcinoma
